# Self-rated health, lifestyle habits and risk assessment in 75-year-old persons attending preventive clinic visits with a nurse in primary health care: a cross-sectional study

**DOI:** 10.1017/S1463423619000136

**Published:** 2019-07-01

**Authors:** Märit Linderholm, Eva Törnvall, Pia Yngman-Uhlin, Katarina Hjelm

**Affiliations:** 1 Primary Health Care Centre Valdemarsvik, Region of Östergötland, Valdemarsvik, Sweden; 2 Doctoral student, Department of Social and Welfare Studies, Linköping University, Norrköping, Sweden; 3 Management Department, Region of Östergötland, and Department of Medical and Health Sciences, Linköping University, Linköping, Sweden; 4 The Research and Development Unit, Region of Östergötland, and Department of Medical and Health Sciences, Linköping University, Linköping, Sweden; 5 Department of Social and Welfare Studies, Linköping University, Norrköping, Sweden; 6 Departement of Public Health and Caring Sciences, Uppsala University, Uppsala, Sweden

**Keywords:** cross-sectional study, lifestyle, preventive clinic visit, primary health care, self-rated health, 75-year-old persons

## Abstract

**Aim:**

To describe self-rated health in relation to lifestyle and illnesses and to identify risk factors for ill health such as pressure ulcers, falls and malnutrition among 75-year-old participants in a new clinical routine involving health assessment followed by tailored one-to-one health promotion at preventive clinic visits to a nurse at primary health care centres (PHCC).

**Background:**

There is a rapidly growing ageing population worldwide. It is central to health policy to promote active and healthy ageing. Preventive clinic visits to a nurse in primary health care were introduced as a new clinical intervention in a region in Sweden to improve the quality of health for the older adults.

**Design:**

A quantitative cross-sectional population-based study.

**Methods:**

The sample consisted of 306 individuals in six primary health care centres in Sweden aged 75 years who attended preventive clinic visits to a nurse. Data were collected from March 2014 to May 2015 during structured conversations with a nurse based on self-administered questionnaires, clinical examinations, risk assessments and after the clinic visit existing register data were collected by the researcher.

**Findings:**

Participants experienced good self-rated health despite being overweight and having chronic illnesses. Daily exercise such as walking and housework was more common than aerobic physical training. The majority had no problems with mobility but reported anxiety, pain and discomfort and had increased risk of falls.

**Conclusion:**

It is important to encourage the older adults to live actively and independently for as long as possible. The healthy older adults may benefit from the clinical intervention described here to support the individual’s ability to maintain control over their health. Such supportive assessments might help the healthy older adult to achieve active ageing, reducing morbidity and preventing functional decline.

## Introduction

The proportion of older people in the population is expected to increase worldwide, but this increase will be more rapid in less-developed regions, according to the United Nations (2013). Decreased birth rates, a sharp drop in mortality and increased life span are contributing to an ageing population in Europe (United Nations, 2013). In Sweden, one fourth of the Swedish population will be older than 65 years in 2050, an increase of 30% (National Board of Health and Welfare, 2010). These changes require strategic planning of cost-effective health promotion interventions through services for the older adults, especially in primary health care.

Preventive home visits to older adults is a concept that describes health promotion activities in the form of visits to the older adults in their homes by a district nurse, aiming at delaying the onset of impairment as long as possible (Vass *et al*., 2007b). Such preventive home visits by district nurses have previously been investigated (Sherman *et al*., 2012). The general aim of these preventive home visits was to describe 75-year-old persons’ self-reported health and health conditions but also to analyse the changes and effects on their health after a preventive visit by the district nurse. The 75-year-old participants rated their health as good or very good and were aware of the benefits of an active lifestyle but they also reported several health problems (Sherman *et al*., 2012). Results from a meta-analysis of studies evaluating the efficacy of various preventive primary care interventions aimed at older adults are to some extent inconclusive (Ploeg *et al*., 2005). These interventions appeared to reduce mortality by 17% and to increase the likelihood of continuing to live in their homes by 23%, but they showed no reduction in admissions to acute care hospitals or long-term care. Costa-de Lima *et al*. (2015) also state that intervention programmes in primary care for healthy older adults can promote health and functionality and reduce mortality and healthcare expenditure and social costs. This review focusing on health in older adults provides only a minimal description of the preventive interventions themselves but often focusing on specific problems such as hypertension, risk of falls and avoiding depression. These studies do not consider the reality of the majority of the older adults, who often present various risk conditions and chronic diseases. The authors state that to have an impact on the quality of life, it is important to perform broader assessments in primary health care. Such broader assessments can be useful for implementing prevention strategies that consider older adults illnesses and complications in a more comprehensive way (Costa-de Lima *et al*., 2015). Previous studies have focused on health in older adults but no study was found focusing on health in older adults attending a preventive clinic visit to a nurse in a primary health care centre (PHCC).

## Background

Normal ageing is characterized by progressive and irreversible changes in both body structure and function (Clegg *et al*., 2013). Improvements in medical care have resulted in many people living longer even with symptoms of chronic diseases (Rosen and Haglund, 2005; Parker and Thorslund, 2007). The most plausible scenario in later life is that the period of disablement and morbidity is condensed towards the end of life (Stepukonis and Svensson, 2006; Fries, 2012). A reduced period of morbidity and a longer time for active and healthy ageing is important to promote active ageing and reduce medical care costs (Gill *et al*., 2010; Fries, 2012).

In Sweden, 70% of people aged 16–84 years rated their health as good or very good. Men rated their health as better than women. The oldest age group (64–84 years) had a somewhat improved self-rated health compared to younger people (Public Health Agency, 2016). The literature on frailty has increased recently but there is a lack of research on the healthy and active older adults although they are in the majority (Friedman *et al*., 2015). Preventive home visits have been used to support health and to facilitate older adults living in their homes as long as possible (Markle-Reid *et al*., 2006; Vass *et al*., 2007a; 2007b; Yamada *et al*., 2012). Home visits often contribute to the development of trusting relationships with those who are healthy and therefore not normally seen in the health care system. They provide an opportunity to talk about the person’s most important needs and focus mainly on health and an active and independent everyday life (Vass *et al*., 2007b; Yamada *et al*., 2012). In a Canadian review, the advantages of performing preventive home visits by a nurse also included decreased mortality, fewer hospital admissions and economic gains (Markle-Reid *et al*., 2006).

The older adults in Sweden had a positive attitude towards home visits by a district nurse and reported good or very good health but also mentioned problems such as anxiety, underweight or overweight, pain, fatigue, sleeping problems and poor understanding of their own health and illness (Sherman *et al*., 2012; 2016). For several years, in Denmark, preventive home visits to older adults have been required by state law. As a result, studies have shown that health promotion decreased functional decline, reduced mortality and the number of admissions to nursing homes (Vass *et al*., 2007a; 2007b; Yamada *et al*., 2012).

Primary health care in Sweden delivers first-line treatment in PHCCs staffed by general practitioners, registered nurses, district nurses, assistant nurses and rehabilitation staff. Nurses provide treatments independently and share the responsibility for patient care with physicians and other health care professionals. The care also includes preventive and educational activities (SOU, 2013). In this study, registered nurses and district nurses are included under the term nurses. In 2012, a local agreement to set up preventive clinic visits to nurses in primary health care was implemented in a region in Sweden. The objective of this study was to describe self-rated health in relation to lifestyle and illnesses and to identify risk factors for ill health such as pressure ulcers, falls and malnutrition among 75-year-old participants in a new clinical routine involving health assessment followed by tailored one-to-one health promotion at preventive clinic visits to a nurse at PHCC.

## Methods

### Study design

A quantitative cross-sectional population-based design was used. Data were collected during structured conversations between the 75-year-old person and a nurse. The person had filled in three self-completed questionnaires before the visit and this served as the basis for the conversation. Clinical examinations and risk assessment were performed during the visit. After the clinic visits, pre-registered data were collected from the medical records by the researcher and noted in a structured form.

### Participants and setting

**Figure 1.**
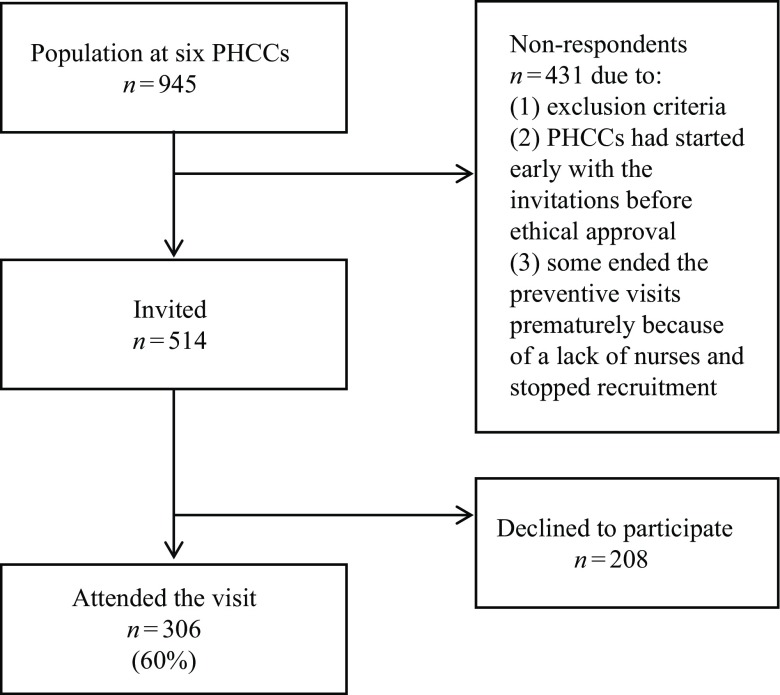
Flowchart of participants and the response rate from March 2014 to May 2015. PHCC, primary health care centre

The study was performed in six PHCCs in the Region of Östergötland in Sweden with a large ageing population selected to obtain a spread across the region. The PHCCs were situated in both cities and provincial communities with between 7300 and 148 000 inhabitants and different socio-economic conditions. In all six areas, preventive clinic visits to a nurse were planned and then took place according to a clinic intervention. All individuals aged 75 years were invited to participate in a preventive clinic visit with a nurse. The management group in the region decided that 75-year-old persons should be offered a clinic visit because the health care centers had already conducted such visits in the population up to 60 years. The idea was that 75-year-olds would still be active and healthy and that they probably had several years to live, and that this would motivate them to participate. The invitation was sent to the person two to four weeks before the visit and the participants did not have to respond to the invitation as this was something they had not asked for. Then they could simply not come for the visit without further explanation. The visit was subject to a fee of 100 SEK (about 9 €). Permission to conduct the study was given by the PHCC managers. The nurses at the PHCCs sent an invitation to those aged 75 years who were registered with the PHCCs. The invitation stated that each individual could attend the preventive office visit exclusively but could also participate in the research project. They were also given written information about the study and asked to give both verbal and written informed consent to participate. Those who were known to have cognitive impairment with difficulties that affected their ability to make decisions as well as patients admitted to palliative home care and who were in a late state of illness were not invited to participate. The total population of 75-year-olds in the six PHCCs was 945 and 514 were invited to participate in the preventive visits. The final sample in this study consisted of 306 (60%) individuals from six PHCCs (Figure 1).

### Data collection

Data collection started in March 2014 and continued until May 2015. In all six areas, preventive clinic visits to a nurse were performed according to a clinical routine. Data were collected, during the clinic visit in structured conversations between the 75-year-old person and a nurse with self-completed questionnaires, clinical examinations and risk assessment. After the clinic visit, pre-registered data were collected from the medical record by the researcher and noted in a structured form.

### Self-administered questionnaires

Data were collected by the nurse during the clinic visit and registered based on three self-completed questionnaires, that were sent out to the participants before the visit together with the invitation: (1) The Health Sheet (Hälsobladet) (2) EQ-5D-5L (3) Self-completed form for medications.

1. A questionnaire The Health Sheet (Hälsobladet) included in the electronic patient record and developed by guidelines from the National Board of Health and Welfare (2011) was used. It includes 11 questions about lifestyle, such as consumption of standard glasses of alcohol per week (i.e., 50 cl beer, 33 cl strong beer, 12–15 cl wine, 8 cl stronger wine or 4 cl spirits); the number of glasses on one occasion; previous or present tobacco use (i.e., never been a smoker/snuff user,have stopped more/less than six months ago, or daily smoker/snuff user; time (min/week) spent on physical training causing shortness of breath (i.e., running, ball sports or gymnastics); time (min/week) spent on daily exercises (i.e., walking, cycling, daily household chores or gardening). The six answer options about physical training were from 0 to more than 120 min per week while the seven options about daily exercise were from 0 to more than 300 min. Finally, eating habits were assessed by questions on how often per week the participants consumed vegetables, fruit, fish and sweets, and how often they ate breakfast.
2. EQ-5D-5L (the Swedish version for Sweden) a standardized translation for measure of health status was used (EuroQol, 1990). It deals with questions on self-rated health with five questions on mobility, personal care, daily activities, pain and discomfort and if the person has feelings of worry or depression. The answer options are scored 1–5 (1, no difficulties walking; 2, slight difficulties walking; 3, moderate difficulties walking; 4, great difficulties walking; 5, can’t walk. The respondents were asked to rate their health on a scale 0–100 where 0 is the worst health you can think of and 100 is the best possible health (EuroQol, 1990).
3. Current prescribed medications were noted by the participant on a self-completed form that was sent out before the visit together with the invitation. During the visit, the nurse then compared this information with the participant’s medical record.

### Clinical examinations

The routine clinical examinations included measurement of blood pressure, weight and height, measured by the nurse during the visit. Blood pressure was measured after 5 to 10 min rest, in the right arm with the person sitting in a chair. Normal systolic blood pressure (SBP) was ≤140 mmHg and normal diastolic blood pressure (DBP) ≤80 mm Hg (World Health Organization, 2017). Body mass index (BMI) was calculated and classified for adults (World Health Organization, 2016a).

### Pre-registered data

Medical diagnoses according to ICD-10 (World Health Organization, 2016b) and how often the participants visited the PHCC were collected by the researcher after the clinic visit from pre-registered health care data in the Region of Östergötland from the date of the preventive visit and three years back in time. Temporary diagnoses such as earwax, upper respiratory tract infection and other minor infections were included in the group other diseases/disorders.

### Risk assessment

During the clinic visit to the nurse at the PHCC, risk assessments for pressure ulcers, falls and nutritional status were made by the nurse. Risk of pressure ulcers was assessed according to the Risk Assessment Pressure Score (RAPS) (Lindgren *et al*., 2002); a questionnaire with 10 questions with three to four answer options on general condition, physical activity, mobility, food and fluid intake and skin condition. A final score ≤29 indicates whether the person has an increased risk of pressure ulcers or not. Risk of falls was assessed with the Downton Fall Risk Index (DFRI) (Downton, 1993); which includes five questions about previous falls, medication, sensory and cognitive impairments and walking ability. A final score ≥3 indicates that the person has an increased fall risk. Finally, malnutrition was assessed with the Mini Nutritional Assessment (MNA) (Vellas *et al*., 2006), which includes six questions on weight loss, loss of appetite, digestive, chewing or swallowing problems, mobility, neuropsychological problems, psychological stress or acute disease. A final score of 8–11 indicates whether the person has a risk of becoming malnourished and a score of 0–7 indicates that the person is already malnourished.

### Data analysis

Data are presented as descriptive statistics. Comparisons between groups were performed by two-tailed Student’s *t*-tests for continuous variables and the Pearson’s chi-square test for categorical variables and when data were on interval level, a nominal scale or not normally distributed (Altman, 1994). *P* < 0.05 was considered statistically significant. The statistical software package SPSS version 22 was used.

## Findings

### Characteristics of the study population

A total of 306 older adults participated in the study, of whom almost 53% were women (Table 1). Most participants lived with a partner. Women more often lived alone than men. BMI values in the population ranged between 18.9 and 46.0 kg/m^2^. More than two thirds of the group were overweight and, of these, one fourth were classified as obese with a BMI > 30 kg/m^2^, none were underweight according to the WHO classification for adults (Table 1). Most had normal blood pressure (SBP ≤ 140 mmHg, 64.5%; DBP ≤ 80 mmHg, 76.2%).

**Table 1.** Characteristics of the study population, marital status, clinical examinations, medical diagnoses, medications and risk factors for ill health (*N* = 306)

	Total, *n* (%)	Men, *n* (%)	Women, *n* (%)	*P* value
Gender		145 (47.4)	161 (52.6)	
Marital status				<0.001
Living in a relationship	220 (79.7)	125 (92.6)	95 (67.4)	NS
Living alone	56 (20.3)	10 (7.4)	46 (32.6)	NS
BMI (kg/m^2^)^a^	27.2 ± 4.5	26.9 ± 3.9	27.6 ± 5.1	0.137
Normal weight	99 (32.7)	46 (32.4)	53 (32.9)	NS
Overweight	130 (42.9)	68 (47.9)	62 (38.5)	NS
Obese	74 (24.4)	28 (19.7)	46 (28.6)	NS
Systolic blood pressure (mm Hg)^b^	137 (17)	133 (18)	140 (16)	<0.001
Diastolic blood pressure (mm Hg)^b^	74 (11)	73 (12)	75 (10)	0.911
Medical diagnoses^c^				
Diabetes mellitus	31 (10.7)	16 (11.6)	15 (9.9)	0.649
Heart disease	122 (42.2)	60 (43.5)	62 (41.1)	0.678
Mental illness	17 (5.9)	7 (5.1)	10 (6.6)	0.576
Respiratory disease	46 (15.9)	19 (13.8)	27 (17.9)	0.340
Arthritis	58 (20.1)	27 (19.6)	31 (20.5)	0.838
Skeletal and muscular diseases	93 (32.2)	34 (24.6)	59 (39.1)	0.009
Tumour diseases	24 (8.3)	14 (10.1)	10 (6.6)	0.278
Other diseases/disorders	226 (78.2)	113 (81.9)	113 (74.8)	0.147
Medications				
Prescribed medications/day				0.144
Mean (SD)	4.46 (3.70)	4.58 (4.05)	4.35 (3.35)	
Range	0–18	0–18	0–16	
No medication	42 (14.4)	22 (15.8)	20 (13.1)	
Type of medication^d^				
Acid regulatory (A02)	51 (17.8)	22 (16.1)	29 (19.3)	0.468
Anticoagulants (B01)	110 (38.3)	67 (48.9)	43 (28.7)	<0.001
Antidepressants (NO6A)	22 (7.7)	7 (5.1)	15 (10.0)	0.120
Antihypertensives (C02)	149 (51.9)	73 (53.3)	76 (50.7)	0.657
Analgesics (N02)	80 (27.9)	36 (26.3)	44 (29.3)	0.564
Asthma/COPD (R03)	36 (12.5)	16 (11.7)	20 (13.3)	0.673
Diabetes treatment (A10)	22 (7.7)	14 (10.2)	8 (5.3)	0.120
Diuretics (C03)	55 (19.2)	26 (19.0)	29 (19.3)	0.939
Intestinal disorder (A03)	13 (4.5)	5 (3.7)	8 (5.3)	0.502
Iron/B_12_/folate deficiency (B03)	39 (13.6)	20 (14.6)	19 (12.7)	0.633
Lipid-lowering (C10)	86 (30.0)	46 (33.6)	40 (26.7)	0.202
Minerals/calcium (A12)	31 (10.8)	8 (5.8)	23 (15.3)	0.010
Ophthalmology (S01)	25 (8.7)	13 (9.5)	12 (8.0)	0.655
Osteoporosis drugs (M05)	10 (3.5)	1 (0.7)	9 (6.0)	0.015
Sedatives/hypnotics (N05)	39 (13.6)	11 (8.0)	28 (18.7)	0.009
Systemic corticosteroids (H02)	17 (5.9)	7 (5.1)	10 (6.7)	0.577
Thyroid (H03)	27 (9.4)	5 (3.6)	22 (14.7)	0.001
Risk factors for ill health^e^				
Pressure ulcers	2 (0.7)	2 (1.4)	0	0.135
Malnutrition	24 (7.9)	11 (7.7)	13 (8.2)	0.877
Falls	80 (26.5)	36 (25.2)	44 (27.7)	0.623

a BMI according to WHO classification for adults 2016.

b Values are mean (SD).

c Medical diagnoses classified with ICD-10.

d Medication are classified according to the generic ATC register in Pharmaceutical Specialities in Sweden (FASS).

e Risk assessments were performed with Risk Assessment Pressure Ulcer (RAPS), Mini Nutritional Assessment and Downton Fall Risk Index (DFRI).

Comparisons between groups were performed using Pearson’s Chi-square test.

Women had higher SBP than men. The most common diagnoses were heart diseases. Fourteen per cent never used any prescribed medications. The mean number of prescribed drugs was five per person/day. Most frequently used were antihypertensive, anticoagulant and lipid-lowering drugs. Women used significantly more sedatives, minerals/calcium, osteoporosis and thyroid drugs than men who used more anticoagulants (Table 1).

### Self-rated health and EQ-5D-5L

Irrespective of gender the participants rated their health as good (Table 2). Patients diagnosed with arthritis (*P* = 0.032), diabetes (*P* = 0.015) and musculoskeletal diseases (*P* = 0.006) rated their health worse than others. Most, 64% had no mobility problems or difficulties with personal self-care and usual daily activities, but 79% had some pain and discomfort. Anxiety and depression expressed as being worried was found in almost 39% of this population. No difference was found between anxiety/depression and gender.

**Table 2.** Self-rated health and EQ-5D-5L dimensions (*N* = 306)

	Total, *n* (%)	Men, *n* (%)	Women, *n* (%)	*P* value
Self-rated health (scale 0–100)^a^	74.5 (18.5)	74.3 (19.4)	74.7 (17.6)	0.487
Mobility^b^				0.559
No problem	192 (64.4)	91 (64.5)	101 (64.4)	
Slight problem	56 (18.8)	25 (17.7)	31 (19.7)	
Moderate problem	38 (12.8)	17 (12.1)	21 (13.4)	
Severe problem	12 (4.0)	8 (5.7)	4 (2.5)	
Unable to walk about	0	0	0	
Self-care^b^				0.437
No problem	267 (89.3)	123 (86.7)	144 (91.8)	
Slight problem	19 (6.4)	10 (7.0)	9 (5.7)	
Moderate problem	11 (3.7)	7 (4.9)	4 (2.5)	
Severe problem	1 (0.3)	1 (0.7)	0	
Unable to wash or dress	1 (0.3)	1 (0.7)	0	
Usual activities^b^				0.137
No problem	197 (66.1)	90 (63.4)	107 (68.6)	
Slight problem	57 (19.1)	24 (16.9)	33 (21.2)	
Moderate problem	32 (10.7)	19 (13.4)	13 (8.3)	
Severe problem	10 (3.4)	7 (4.9)	3 (1.9)	
Unable to do usual activities	2 (0.7)	2 (1.4)	0	
Pain/discomfort^b^				0.267
No pain/discomfort	63 (21.1)	36 (25.4)	27 (17.2)	
Slight pain/discomfort	131 (43.8)	55 (38.7)	76 (48.4)	
Moderate pain/discomfort	88 (29.4)	43 (30.3)	45 (28.7)	
Severe pain/discomfort	16 (5.4)	7 (4.9)	9 (5.7)	
Extreme pain/discomfort	1 (0.3)	1 (0.7)	0	
Anxiety/depression^b^				0.100
Not anxious or depressed	184 (61.6)	96 (67.6)	88 (56.1)	
Slightly anxious or depressed	90 (30.1)	33 (23.2)	57 (36.3)	
Moderately anxious or depressed	21 (7.0)	12 (8.5)	9 (5.7)	
Severely anxious or depressed	3 (1.0)	1 (0.7)	2 (1.3)	
Extremely anxious or depressed	1 (0.3)	0	1 (0.6)	

a Mean (SD).

b Frequency of reported problems in each dimension of EQ-5D-5L (Swedish version).

Comparisons between groups were performed using Pearson’s chi-square test.

### Risk factors for ill health

Almost all of the participants were mobile and lived an active life and only two were at risk of pressure ulcers (Table 1). Twenty-four persons were at risk of developing malnutrition and one person was malnourished according to the risk assessment in MNA. More than one fourth of the population had an increased fall risk. A significant difference was found between fall risk and pain (*P* = 0.004), anxiety (*P* = 0.001), mobility (*P* ≤ 0.001) and daily exercise (*P* = 0.001). This showed that those with more pain, more anxiety, less mobility and less daily exercise had a greater fall risk. There was no significant difference between the risk of falling and those who did physical training causing shortness of breath less or more.

### Lifestyle habits

#### Dietary habits and physical activity

More than two thirds ate vegetables once a day or more, and women consumed significantly more vegetables than men (Table 3). Most of the group consumed fruit once a day or more, and women ate fruit more often every day than men. Half of the population ate fish and seafood once a week and nearly half of them consumed sweets once to twice a day or more. Sixty-four percent of the participants did not exercise at all or performed weekly physical training causing shortness of breath, that is, running, ball sports or gymnastics less than 30 min/week. Daily exercise, that is, walking, cycling, daily household chores or gardening, was more common, and more than two thirds of the population performed such activity for more than 90 min/week. Persons who were overweight exercised daily to a greater extent than those of normal weight and obese individuals (*P* = 0.035). There was also a significant difference between daily exercise and mobility (*P* ≤ 0.001), those who exercised most had least problems with mobility, and between daily exercise and pain (*P* = 0.026), those with severe or extreme pain exercised daily to a lesser extent. A significant difference was also found between daily exercise and anxiety; persons who were more worried tended to exercise to a lesser extent than those with less anxiety (*P* ≤ 0.001).

**Table 3.** Lifestyle habits of the participants (*N*=306)

	Total, *n* (%)	Men (*n* = 145)*, n* (%)	Women (*n* = 161), *n* (%)	*P* value
How often do you eat vegetables?				**<**0.001
A few times/week or less	96 (31.6)	61 (42.0)	35 (21.8)	
Once every day	162 (53.3)	70 (48.3)	92 (57.1)	
Twice a day or more	46 (15.1)	12 (8.3)	34 (21.1)	
How often do you eat fruit?				0.076
A few times/week or less	56 (18.6)	32 (22.0)	24 (14.9)	
Once every day	134 (44.4)	71 (49.0)	63 (39.1)	
Twice a day or more	112 (37.1)	40 (27.6)	72 (44.7)	
How often do you eat fish?				0.153
A few times/month or less	22 (7.3)	15 (10.3)	7 (4.3)	
Once a week	169 (55.8)	71 (49.0)	98 (60.9)	
Two times/week or more	112 (36.9)	58 (40.0)	54 (33.5)	
How often do you eat sweets, pastries, snacks, soft drinks?				0.282
A few times/week or less	156 (51.1)	71 (49.0)	85 (52.8)	
Once a day	125 (41.0)	58 (40.0)	67 (41.6)	
Twice a day or more	24 (7.9)	15 (10.3)	9 (5.6)	
Do you have breakfast?				0.551
A few times/week or less	8 (2.6)	2 (1.4)	6 (3.8)	
Nearly every day	5 (1.6)	3 (2.1)	2 (1.2)	
Every day	292 (95.8)	139 (95.9)	153 (95.0)	
Physical training/week (ball sports, running, gymnastics)				0.053
No training or <30 min	193 (64.1)	93 (64.1)	100 (70.4)	
30–120 min	86 (28.6)	35 (24.1)	51 (31.6)	
>120 min	22 (7.3)	12 (8.3)	10 (6.2)	
Daily exercise/week (walking, cycling, gardening)				0.182
No exercise or <30 min	23 (7.8)	13 (9.0)	10 (6.2)	
30–150 min	127 (42.8)	54 (37.3)	73 (45.3)	
>150 min	147 (49.5)	72 (49.6)	75 (46.6)	
Standard drinks/week				**<**0.001
<1 glass	159 (52.5)	57 (39.3)	102 (63.4)	
1–4 glasses	104 (34.3)	53 (36.6)	51 (31.7)	
5–9 glasses	31 (10.2)	26 (17.9)	5 (3.1)	
10–14 glasses	9 (3.0)	8 (5.5)	1 (0.6)	
Standard drinks on a single occasion				0.087
Never	215 (71.2)	91 (62.8)	124 (77.0)	
Less than once/month	57 (18.9)	35 (24.1)	22 (13.7)	
Every month	16 (5.3)	9 (6.2)	7 (4.3)	
Every week	12 (4.0)	6 (4.1)	6 (3.7)	
Smoking				0.012
Never smoked	167 (54.6)	63 (43.4)	104 (64.6)	
Previous smoker	123 (40.7)	73 (50.4)	50 (31)	
Present smoker	15 (4.9)	8 (5.6)	7 (4.3)	
Snuff user				**<**0.001
Never been a snuff user	275 (90.2)	115 (79.3)	160 (99.4)	
Previous snuff user	14 (4.6)	14 (9.7)	0 (0.0)	
Present snuff user	16 (5.2)	15 (10.4)	1 (0.6)	

Comparisons between groups were performed using the Pearson’s chi-square test.

#### Alcohol and tobacco use

More than half of the participants used no alcohol at all or drank less than one standard glass/week, but overall there was a significantly higher consumption among men than women (Table 3). Half of the study population had never smoked, and less than 5% were daily smokers. Most, 65% of the women had never smoked. More than 90% of the study population had never been snuff users, but the majority of daily snuff users were men.

## Discussion

The main results showed that the participants rated their health as good or very good and lived an active life, although they were overweight, reported anxiety, pain and discomfort, had chronic diseases and an increased risk of falls.

The rating of health as good or very good in this study was consistent with the results for a Swedish population where more than 70% of the group (16–84 years) answered that their health was good or very good (Public Health Agency, 2016). Self-rated health was measured on a five-point scale as agreed by the European Union. Men reported better health than women and the results for the oldest age group (64–84 years) showed that they had somewhat improved self-rated health compared to younger people (Public Health Agency, 2016). In our study, there were no gender differences in self-rated health. Although the participants rated their health highly, they also reported pain, discomfort and feelings of anxiety. The older population often have preserved functional status but are also diagnosed with chronic medical conditions (Gill *et al*., 2010: Fries, 2012). Today, postponing functional decline and compressing morbidity into a later period in life is more important than focusing on reducing mortality. These are important theoretical approaches to both improved health and reduction in the cost of medical care (Gill *et al*., 2010; Fries, 2012). There are few older adults totally free of age-associated disease and significant physiologic deterioration (Beard *et al*., 2015) but the really frail are still a minority of older adults (Friedman *et al*., 2015). The participants in our study lived an active life, although they also had different symptoms and chronic diseases. Advances in medical treatment have successfully reduced the impact of chronic diseases on health and functional capacity, and this to some extent explains the positive health developments in the older adults (Friedman *et al*., 2015; Costa-de Lima *et al*., 2015). It therefore seems appropriate, both for primary health care and the geriatric community, to promote healthy ageing in older adults with preserved function and few chronic medical conditions before the onset of frailty (Friedman *et al*., 2015).

A Cochrane report has shown that general health checks in healthy adults do not reduce the risk of morbidity or mortality; instead, there was an increase in the number of diagnoses (Krogsbøll *et al*., 2012). However, although this report is often cited by health care policy it may be misleading when it comes to health checks in the older population because this Cochrane report excluded trials targeting older persons aged >65 years. Preventive interventions might postpone functional decline and independence, which in a larger context can reduce the cost of health care but maybe more importantly can increase the well-being and quality of life of the individual. It is a challenge to design care initiatives that can reach the groups with the greatest need. To organize primary care targeted at the older adults in established senior consultations and in close corporation with the municipalities could create an opportunity to provide holistic and person-centred care.

Almost two thirds of our population were classified as overweight and no one was underweight according to the BMI classification for adults (World Health Organization, 2016a). Our findings reveal a discrepancy between the MNA assessment whereby one person was malnourished and nearly 8% were at risk of malnutrition, and the BMI values, which were classified according to the WHO, whereby no one was underweight. The WHO BMI classification is a rough guide, which may not perfectly correspond to older adults; the older population are at risk of age-related misclassification with underestimation of malnutrition and an overestimation of the number of who are overweight (Sanchez-Garcia *et al*., 2007; Gavriilidou *et al*., 2015).

One fourth of the population had an increased fall risk. There was significant difference between fall risk and pain, anxiety, mobility and daily exercise. But no significant difference was found between fall risk and physical training. Today, there is consensus that falling and having a low BMI are associated with an increased risk of hip fractures among older adults. The greatest risk is seen in those who have lost weight, regardless of the intent behind the weight loss, particularly in women (Ensrud *et al*., 2003). It seems important to screen for fall risk even in well-functioning healthier older persons because falls are the leading cause of nonfatal and fatal injuries among older adults and are a major threat to health and independence (Ambrose *et al*., 2015).

The current intervention, along with the selection of questionnaires to be used, was planned by the management group in the region and was decided without the researchers’ involvement; to show the physical activity level more clearly, it might have been more appropriate for this age-group to use other validated instruments rather than the questionnaire The Health Sheet (Hälsobladet) used in this intervention. Examples of instruments that could have been used are the International Physical Activity Questionnaire (IPAQ), an instrument that mainly applies to healthy older adults and focuses on leisure activities (Ekelund *et al*, 2006) or Physical Activity Scale for Elderly (PASE), a measuring instrument with more response levels which contains questions about sedentary time, household activities and recreational activities (Washburn *et al*, 1999).

In today’s literature, physical activity is directed at older adults and is regarded as the most important preventive measure that has an impact on the person’s quality of life, function, independence and health. Older adults can improve strength, balance and flexibility as well as their aerobic fitness in advanced age. The current recommendation is that persons with chronic illness or disability should be as active as possible (Swedish National Institute of Public Health, 2017). Exercise that improves strength and balance is seen as the most important intervention that can prevent falls in healthy older adults (Beswick *et al*., 2008; Sherrington *et al*., 2016). The fact that the participants in our study had problems such as musculoskeletal diseases and reported both pain and anxiety may have contributed to their difficulty participating in physical activities that caused shortness of breath. In this study, there were also significant differences between exercising every day and anxiety and the use of antidepressive medication. It may be that those with depressive symptoms did not have the energy and initiative to start such activities on their own. Several studies have shown that this can be an important task for health care professionals to promote physical exercise tailored to individual ability to reduce the severity of depressive symptoms (Bridle *et al*., 2012; Heinzel *et al*., 2015).

The public health approach should be towards preserving both physical and mental functions, disease and injury prevention, for example, from falls among older adults. It must simply be a matter of priority at all levels in the organizations.

### Strengths and limitations

The strength of this study was the population-based study design, with a sampling technique that involved a total population provided they met the inclusion criteria. Persons with mild cognitive impairment were excluded from participating in the study. It can be a problem that older adults with mild cognitive impairment too often are excluded from participating in research as they make up a significant part of the population of 75-year-olds. Future research could include these participants if their caregivers are also included in the study.

It was a challenge to maintain the continuity in the data collection since the study was a clinical multi-centre study involving many nurses and also was extended over a long period of time. However, the respondent rate was almost 60%, which is consistent with studies in primary health care (Abramson and Abramson, 2008) and consistent with the study from Denmark, where 60% accepted the home visit (Vass *et al*, 2007b). A limitation was some missing data in the documentation about marital status and earlier occupation. This is usually documented in the medical record and the nurses may therefore have omitted to ask about that.

Moreover, some of the 75-year-olds in our study did not attend the visit and there is a lack of information on why they declined to attend the preventive visit at PHCC. Possibly, it was just those who were mobile who chose to participate, or that the participants had to pay a visit fee. It may also be that they felt too healthy, too busy or too ill to participate. Those who agree to participate may have different motivations or life circumstances than those who do not (Polit and Beck, 2013).

### Implications for health care and research

The results suggest that the fall risk and feelings of anxiety and pain/discomfort among this older population should encourage health care professionals to increase their work on measures to prevent falls and identify and support those with pain and anxiety. When using preventive nursing interventions such as clinic visits to a nurse in primary health care, it is crucial that these interventions are adapted to older adults’ needs. Health promotion directed towards physical, psychological and social aspects are important and could prove to be an effective component of a public health approach. The clinic visit aimed at 75-year-old persons who are still independent gives the nurse an opportunity to work in a proactive way. The nurse can provide person-centered information and help with referrals to other public services, examine untreated health problems and promote medication compliance. This can help the elderly to prevent functional impairment and to establish a relationship with staff in primary health care to be used in the future if needed.

Compression of morbidity and prolonging active and healthy ageing are central to today’s health policy issues and an important theoretical approach. Preventive home visits by district nurses have been investigated but no studies have focused on health in older adults attending clinic visits to a nurse in a primary health care centre. It is important for primary health care to support older adults with preventive efforts to maintain healthy ageing, despite age-related and chronic diseases, to extend the period of well-being and independence before the onset of frailty. There is a lack of research on health strategies in primary health care oriented towards the healthy older adults who are still independent.

## Conclusion

From this study, we suggest that healthy older adults can benefit from the clinical routine described; preventive conversations with a nurse can support the individual’s ability to maintain and increase their control over their health and to achieve active ageing, reducing morbidity and preventing functional decline for as long as possible. Nevertheless, further research is needed to find ways to reach the older adults with the greatest need and who choose not to participate in offered preventive activities.
